# Detecting insomnia in patients with low back pain: accuracy of four self-report sleep measures

**DOI:** 10.1186/1471-2474-14-196

**Published:** 2013-06-27

**Authors:** Saad M Alsaadi, James H McAuley, Julia M Hush, Delwyn J Bartlett, Nicholas Henschke, Ronald R Grunstein, Chris G Maher

**Affiliations:** 1 The George Institute for Global Health, Sydney Medical School, University of Sydney, Missenden Road, P.O. Box M201, Sydney, New South Wales, Australia; 2 Neuroscience Research Australia and the University of New South Wales, Sydney, New South Wales, Australia; 3 Department of Health Professions, Macquarie University, Sydney, New South Wales, Australia; 4 The Woolcock Institute of Medical Research, University of Sydney, Sydney, New South Wales, Australia; 5 Institute of Public Health, University of Heidelberg, Heidelberg, Germany; 6 Department of Respiratory and Sleep Medicine, Royal Prince Alfred Hospital, Sydney, New South Wales, Australia

**Keywords:** Low back pain, Insomnia, Diagnosis, Questionnaire, Accuracy

## Abstract

**Background:**

Although insomnia is common in patients with low back pain (LBP), it is unknown whether commonly used self-report sleep measures are sufficiently accurate to screen for insomnia in the LBP population. This study investigated the discriminatory properties of the Pittsburgh Sleep Quality Index (Pittsburgh questionnaire), Insomnia Severity Index (Insomnia index), Epworth Sleepiness Scale (Epworth scale) and the sleep item of the Roland and Morris Disability Questionnaire (Roland item) to detect insomnia in patients with LBP by comparing their accuracy to detect insomnia to a sleep diary. The study also aimed to determine the clinical optimal cut-off scores of the questionnaires to detect insomnia in the LBP population.

**Methods:**

Seventy nine patients with LBP completed the four self-reported questionnaires and a sleep diary for 7 consecutive nights. The accuracy of the questionnaires was evaluated using Receiver Operator Characteristic (ROC) curves with the Area Under the Curve (AUC) used to examine each test’s accuracy to discriminate participants with insomnia from those without insomnia.

**Results:**

The Pittsburgh questionnaire and Insomnia index had moderate accuracy to detect insomnia (AUC = 0.79, 95% CI = 0.68 to 0.87 and AUC = 0.78, 95% CI = 0.67 to 0.86 respectively), whereas the Epworth scale and the Roland item were not found to be accurate discriminators (AUC = 0.53, 95% CI = 0. 41 to 0.64 and AUC = 0.64, 95% CI = 0.53 to 0.75 respectively). The cut-off score of > 6 for the Pittsburgh questionnaire and the cut-off point of > 14 for the Insomnia index provided optimal sensitivity and specificity for the detection of insomnia.

**Conclusions:**

The Pittsburgh questionnaire and Insomnia index had similar ability to screen for insomnia in patients with low back pain.

## Background

The International Classification of Sleep Disorders, second edition (ICSD-2), defines insomnia as the complaint of one or more of the following: difficulty in initiating sleep; difficulty in maintaining sleep; waking up too early and unable to resume sleep or nonrestorative sleep, despite adequate preparation for sleep. These symptoms are to be associated with at least one or more forms of day-time functional impairment related to nocturnal sleep difficulty, such as fatigue; mood disturbance; or sleep dissatisfaction [1,2].

Insomnia is considered to be the most frequently occurring sleep disorder [3]. It is often associated with medical conditions such as depression, anxiety and respiratory disorders [4]. Insomnia is also common in painful conditions; recent studies have reported that at least 50% of patients with low back pain (LBP) also report symptoms of insomnia [5,6]. Insomnia is often associated with fatigue, cognitive impairment and mood disturbance, leading to functional impairment [7]. In addition, there is substantial evidence that insomnia can adversely influence an individual’s experience of pain through increasing perception of pain and decreasing pain tolerance and pain threshold [8]. Recent studies have found insomnia symptoms in patients with LBP are significantly associated with pain intensity, day-time functional impairment and psychological distress [5,6,9]. These findings imply that insomnia is likely to adversely affect LBP management. Despite this, there is little published information on which is the most accurate and practical method for assessing insomnia in the LBP population. Clinicians and researchers need accurate, brief and cost-effective measures to screen for insomnia and evaluate treatment outcome.

Self-reported questionnaires are a common method used to assess insomnia as sleep data can be collected from multiple nights, they are brief, easy to administer and cost-effective. Several self-reported questionnaires have been developed to assess aspects of sleep quality and are commonly used to assess insomnia symptoms. These include the Pittsburgh Sleep Quality Index (Pittsburgh questionnaire) [10], Insomnia Severity Index (Insomnia index) [11] and the Epworth Sleepiness Scale (Epworth scale) [12]. Specifically for LBP populations, researchers and clinicians use sleep item(s) of LBP functional questionnaires to assess their patients’ sleep quality. For example, the sleep item of the Roland and Morris Disability Questionnaire (Roland item) [13]. Some of these questionnaires have been translated to different languages and validated for use in several medical conditions such as cancer [14,15] and traumatic brain injury [16].

These questionnaires are potentially useful tools to detect insomnia in patients with LBP. However, their capacity to detect insomnia in this population is currently unknown. It has been suggested that questionnaire’s accuracy to detect insomnia and the optimal cut-off score may differ based on the studied population [14]. In fact, previous clinometric testing of these instruments showed inconsistency for the optimal cut-off scores between different medical conditions as well as community samples [17-20]. Therefore, evaluation of the capacity of these questionnaires to detect insomnia in patients with LBP is necessary before their employment in clinical and research practice.

The subjectivity of the insomnia definition and the absence of a gold standard to diagnose insomnia make it difficult to examine the criterion-related validity of self-reported questionnaires [19]. A solution may be obtained by investigating the sensitivity and specificity of the questionnaire to detect insomnia [19]. Sleep diaries are daily subjective reports of sleep that are widely used to study sleep disturbance [11,21]. They are completed immediately after arising to provide an evaluation of the previous night’s sleep quantity and sleep disturbance, which minimizes recall bias [3]. Although diaries may not be an optimal method for diagnosing certain insomnia subtypes, they are considered to be useful measures to detect general insomnia disorder [1,3,7].

In the current study, we aimed to investigate the properties of four self-reported sleep questionnaires to detect insomnia disorder in patients with LBP by comparing their accuracy to detect insomnia to a reference standard, the Sleep Diary [21]. The study also aimed to determine the optimal cut-off scores of the questionnaires to detect insomnia in the LBP population. We expected that a measure which assesses symptoms of general insomnia, sleep difficulties and day-time impairment (ICSD-2) [1] would accurately detect insomnia and therefore is a suitable reference standard.

## Methods

### Study overview

This was a cross-sectional study conducted between March 2010 and June 2012. The study protocol was approved by the University of Sydney Human Research Ethics Committee, Australia (09-2009/12100). All participants signed an informed consent form before participating in the study. Participants were compensated for their time and transportation expenses.

### Participants

Participants were recruited from physiotherapy clinics in the Sydney metropolitan area, through advertising using flyers and posters in physiotherapy clinics and community centers and by advertising on local social media web sites. The inclusion criteria were: patients with non-specific low back pain (i.e. low back pain with no specific pathology) [22], aged between 18 and 79 years and possessing sufficient fluency in the English language to answer self-completed questionnaires. Exclusion criteria were: sciatica (i.e. pain radiating below the knee with definite neurological signs); spinal surgery within the preceding 6 months; previously diagnosed with a sleep disorder for which they were receiving care; receiving care for a mental health condition; and rotating night shift workers. There was no restriction on duration of LBP.

### Procedures

Recruiting physiotherapists informed patients about the study and passed their contact details to the study researcher. Potential participants from the community were provided with information about the study through the post or email. Volunteers who showed an interest in participating were then contacted and screened for inclusion by the study researcher.

All those who met the eligibility criteria were given an appointment to meet the study researcher at the sleep clinic of the Woolcock Institute of Medical Research, the University of Sydney, Australia. During their visit to the clinic, participants completed a baseline assessment booklet, which took approximately 30 to 45 minutes. The baseline assessment booklet contained:

(i) Demographic questions regarding a participant’s age, gender, weight, height, educational level, employment status, smoking status, and whether the participant was seeking care for LBP or taking medication.

(ii) Self-reported measures of sleep quality, including the Pittsburgh questionnaire, Insomnia index, and Epworth scale.

(iii) The Brief Pain Inventory, which measures pain intensity [23]; the Depression, Anxiety and Stress Scale (DASS-21) [24], which measures depression, anxiety and stress; the Roland and Morris Disability Questionnaire [13], which measures disability due to LBP; and the Fatigue Severity Scale [25], which measures fatigue.

Participants also completed the Pittsburgh sleep diary [21], over 7 consecutive days. Participants were followed up, at least once, either via phone calls or through SMS text messages to ensure that the sleep diary was being completed.

### Measures

#### The Pittsburgh sleep diary (sleep diary)

The sleep diary [21] consists of two sections. Although both sections were completed, only the second section was used in this study. The section collects information about the previous night’s sleep and is completed immediately after awaking. It contains the following items: (1) time went to bed, (2) lights out time, (3) sleep onset latency (SOL) calculated as the minutes from lights out until falling asleep, (4) time of final waking, (5) method/ reason of final waking, (6) number of times the participant woke during the night, wake after sleep onset (WASO), (7) duration of WASO in minutes calculated as the total number of minutes of awake that occurred after sleep onset and before the final awaking, (8) reason(s) for WASO, (9) sleep quality, (10) mood on final awaking, and (11) alertness on final waking. Items 9 to 11 were completed on a 0–10 Numerical Rating Scale (NRS).

#### The Pittsburgh sleep quality index (Pittsburgh questionnaire)

The Pittsburgh questionnaire is a self-report instrument designed to evaluate sleep quality over the last month. It consists of 19 items to produce 7 aspects of sleep quality (sleep onset latency, sleep duration, efficiency, quality, disturbances, medication, and day-time dysfunction). The sum of these 7 aspects (0–3) yields a global score of sleep quality (0–21); a high sore is an indication of poor sleep quality. The cut-off score of > 5 has been found to be an accurate cut-off score to distinguish between patients with primary insomnia and those without insomnia [18].

#### The insomnia severity index (Insomnia index)

The Insomnia index is a 7-item scale, with each item rated on a 5-point Likert-scale. It assesses insomnia severity, sleep satisfaction, sleep interference with day-time functioning, noticeability of sleep impairment, and distress caused by insomnia over the last 2 weeks. Summation of the 7-items provides a score ranging from 0 to 28, where 0–7 indicates no significant insomnia, 8 to 14 indicates sub-threshold insomnia, 15 to 21 indicates moderate insomnia, and 22 to 28 indicates severe insomnia. The cut-off score of > 14 has been reported to be the most accurate point to detect patients with primary insomnia [19].

### The Epworth sleepiness scale (Epworth scale)

The Epworth scale is an 8-item self-report questionnaire used to assess excessive day-time sleepiness over the last week. Participants indicate on a 4-point Likert- type scale (0 = never, 3 = high chance) the likelihood that they will “doze off or fall asleep” in eight different situations. Summation of the 8 responses produces a total score ranging from 0 to 24; with higher scores reflecting greater sleepiness. The cut off score of > 10 has been found to accurately determine excessive day-time sleepiness [26].

#### The sleep item of the Roland and Morris disability questionnaire (Roland item)

The Roland and Morris Disability questionnaire [13] is a 24-item self-administered questionnaire designed to measure the effect of LBP on a patient’s normal activities of daily living. The sleep item (Roland item) examines sleep quality in relation to the effect of pain: “*I sleep less well because of my back*”. The response format of the item is dichotomous (yes/no). The item has been reported to be easy to understand and answer by patients with LBP [27].

The four self-reported sleep questionnaires, the Pittsburgh questionnaire; Insomnia index; Epworth scale and the Roland item were selected for several reasons. Both the Pittsburgh questionnaire and the Insomnia index assess several aspects of insomnia, including sleep difficulty and day-time impairments related to night’s sleep, and have been widely used in the assessment of sleep quality in patients with LBP [28]. On the other hand, a questionnaire with a single item may potentially be easier to complete, less time consuming and easier to score and interpret than a measure with multiple insomnia items [29]. We, therefore, attempted to evaluate the discriminatory properties of the Roland item and the Epworth scale to detect insomnia in patients with LBP. The Roland item assesses sleep difficulty and is commonly employed by clinicians and researchers in the domain of LBP [30] and the Epworth scale is also a common measure that has been translated into 52 languages to assess individual’s day-time sleepiness [31].

#### Insomnia classification

We adopted the general criteria of the International Classification of Sleep Disorder, second edition (ICSD-2) to classify insomnia [1]. Participants were classified as having insomnia if they reported in the sleep diary, for at least 3 of the 7 nights, either (i) wake after sleep onset (WASO) > 30 minutes or (ii) sleep onset latency (SOL) > 30 minutes. These symptoms should be associated with either poor sleep quality or low mood. Poor sleep quality was defined as < 5 on a 0–10 scale where 0 = very bad sleep quality and 10 = very good sleep quality. Low mood was defined as < 5 on final waking on 0–10 scale where 0 = very tense and 10 = very calm. We chose the frequency of ≥ 3 nights and duration of SOL and WASO of > 30 minutes as these are the most commonly recommended criteria [1,3,32].

#### Statistical Analyses

Statistical analyses were conducted as described below using SPSS version 19 (SPSS Inc., Chicago, IL), MedCalc for Windows, version 12.2.1.0 (MedCalc Software, Mariakerke, Belgium) and Meta-DiSc data analysis software [33].

#### Assessment of test properties

Discriminatory properties of self-report questionnaires were tested using the sleep diary as the reference test and each questionnaire as the index test.

1. The scores from the index tests were used to construct Receiver Operator Characteristic (ROC) curves using non-parametric methods [34], with the Area Under the Curve (AUC) used to examine each test’s accuracy to discriminate participants with insomnia from those without insomnia. The AUC ranges from 0 to 1.0 with a value of 0.5 representing discrimination no better than chance. Using DeLong’s method ROC curves were compared to test for statistically significantly differences between AUCs [34]. AUC values were interpreted using guidelines provided by Swets (1988) [35]: (0.5 to 0.7 = low accuracy, 0.7 to 0.9 = moderate accuracy, >0.9 = high accuracy).

2. Index test scores were dichotomised using both the ROC technique and scores based on the literature: for the Pittsburgh questionnaire we used cut-off points of > 5 [18] and > 10 [36], a cut-off > 14 for the Insomnia index [19], and for the Epworth scale we used a cut-off point of >10 [26]. A 2 x 2 table was created to calculate sensitivity, specificity, positive likelihood ratio and negative likelihood ratio. Sensitivity was defined as the proportion of people with insomnia who tested positive, while specificity was defined as the proportion of people without insomnia who tested negative.

## Results

### Participants’ description

A total of 101 patients with LBP were interested in participating in the study. Of those, eighty patients met the study criteria and were enrolled in the study. One participant did not complete the sleep diary and was therefore excluded from the analyses, leaving a total of 79 participants. Participants’ demographic and clinical information are presented in Table 1.The mean Pittsburgh questionnaire score (7.9) was above the cut-off score for poor sleep quality (> 5) [18], while the mean of the Insomnia index total score (11.2) was below the cut-off score of clinical insomnia (i.e. > 14) [19], indicating sub-threshold insomnia. The mean score of the Epworth scale (7.2) was within the normal range (i.e. < 10) [26], an index of normal day-time sleepiness. According to the Roland item 46/79 (58%) participants reported that they sleep less well because of their low back pain. The disability assessment as measured by the Roland and Morris disability questionnaire indicated a moderate level of disability. Scores of psychological distress on the DASS-21 showed levels of depression, stress and anxiety to be within the normal range. Fatigue assessment using the fatigue severity scale showed normal fatigue level. The sleep diary reports showed that none of the participants used a medication that can influence sleep, including insomnia medication, during the study period.

**Table 1:** Sample’s demographic and clinical characteristics

		***Mean (SD)***
Age	(Year)	43.91 (15.4)
Pain intensity^*^	(0–10)	4.11 (1.9)
Physical disability^**^	(0–24)	8.78 (5.3)
Depression^***^	(0–42)	8.75 (9.9)
Anxiety^≠^	(0–42)	6.55 (8.4)
Stress^±^	(0–42)	13.08 (9.8)
Fatigue^†^	(1–63)	32.49 (12.3)
Pittsburgh questionnaire^#^	(0 – 21)	7.92 (3.8)
Insomnia index^$^	(0 – 28)	11.25 (6.4)
Epworth scale^¥^	(0 – 24)	7.26 (5.2)
Body mass index	(Kg/m^2^)	25.73 (4.9)
		***N (%)***
Pain duration	Acute	32 (41%)
	Chronic	47 (59%)
Roland item^£^	(Yes)	46 (58%)
Seeking care	(Yes)	58 (73%)
Taking pain medication	(Yes)	35 (44%)
Gender	(Female)	40 (51%)
Level of education	(University degree)	35 (44%)
Smoking status	(Not)	74 (94%)
Work status	Full-time	48 (61%)
	Part-time	14 (18%)
	Not working	4 (5%)
	Retired	13 (16%)
Marital status	Married or defacto	39 (49%)
	Separated	8 (10%)
	Single	32 (41%)

^*^Brief Pain Inventory on 0–10 NRS, 0 = no pain, 10 = pain as bad as you can imagine. ^**^Roland and Morris Disability Questionnaire: the higher score indicates more severe disability. ^***^Depression subscale of the DASS-21: 0 – 9 = no depression, 10 – 13 = mild depression, 14 – 20 = moderate depression, 21 – 27 = severe depression and over 28 = extremely severe depression. ^≠^Anxiety subscale of the DASS-21: 0 – 7 = no anxiety, 8 – 9 = mild anxiety, 10 - 14 = moderate anxiety, 15 – 19 = severe anxiety and more than 20 = extremely severe anxiety. ^±^Stress subscale of the DASS-21: 0 – 14 = normal level of stress, 15 – 18 = mild stress, 19 – 25 = moderate stress, 26 – 33 = severe stress and over 37 = extremely severe stress. ^†^Fatigue Severity Scale: a total score of less than 36 suggests no fatigue, while a total score of 36 or more suggests fatigue and further evaluation by a physician is needed; ^#^Pittsburgh sleep quality index: > 5 indicates poor sleep quality; ^$^Insomnia severity index: > 14 indicates clinical insomnia; ^¥^Epworth sleepiness scale: > 10 indicates excessive day-time sleepiness.

^£^ Roland item = I sleep less well because of my back.

### Accuracy of questionnaires in insomnia detection

**Figure 1:**
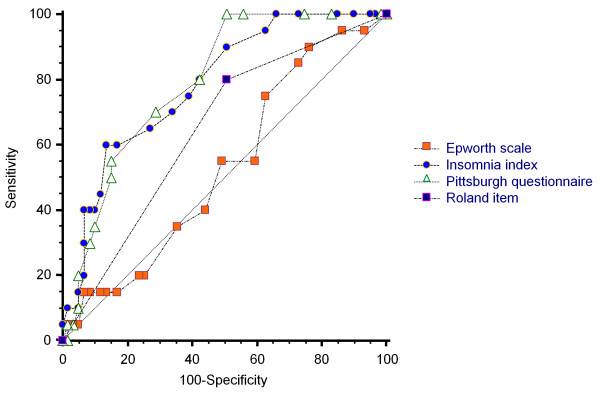
**Receiver operator characteristic (ROC) curves for all sleep measures.** Pittsburgh questionnaire: Pittsburgh sleep quality index, Insomnia index: Insomnia severity index, Roland item: Sleep item of the Roland and Morris disability questionnaire, Epworth scale: Epworth sleepiness scale

Figure 1 and Table 2 illustrate the receiver operator characteristic (ROC) curves and the area under the curves (AUCs) of the four sleep measures. The Pittsburgh questionnaire (AUC = 0.79, 95% CI = 0.68 – 0.87) and Insomnia index (AUC = 0.78, 95% CI = 0.67 – 0.86) showed moderate accuracy in distinguishing between patients with insomnia and those without. In contrast, the Roland item (AUC = 0.64, 95% CI = 0.53 – 0.75) and Epworth scale (AUC = 0.53, 95% CI = 0.41 – 0.64) had only low accuracy. Pairwise comparison between AUCs, using the DeLong method [34], showed that both the Pittsburgh questionnaire and the Insomnia index were significantly different from the Roland item and Epworth scale (p < 0.05) (Table 3).

**Table 2:** Area under the curve of the four measures

***Test***	***AUC***^***a***^	***95% CI***
Pittsburgh questionnaire^b^	0.79	0.68 to 0.87
Insomnia index^c^	0.78	0.67 to 0.86
Roland item^d^	0.64	0.53 to 0.75
Epworth scale^e^	0.53	0.41 to 0.64

^a^ Area under the curve, ^b^ Pittsburgh sleep quality index, ^c^ Insomnia severity index, ^d^ Sleep item of the Roland and Morris disability questionnaire, ^e^ Epworth sleepiness scale.

**Table 3:** Pairwise comparison between areas under the ROC curve

***Comparison***	***Difference between areas***	***z statistic***	***P-value***
	(95% CI)^*^		
Pittsburgh questionnaire^a^ vs Insomnia index^b^	0.01 (−0.07 to 0.09)	0.18	0.85
Pittsburgh questionnaire^a^ vs Roland item^c^	0.14 (0.01 to 0.28)	2.11	0.03
Pittsburgh questionnaire^a^ vs Epworth scale ^d^	0.26 (0.09 to 0.41)	3.03	0.002
Insomnia index^b^ vs Roland item^c^	0.13 (0.003 to 0.27)	2.00	0.04
Insomnia index^b^ vs Epworth scale^d^	0.25 (0.09 to 0.41)	3.05	0.002
Epworth scale^d^ vs Roland item^c^	0.11 (−0.05 to 0.28)	1.33	0.18

^*^Confidence interval, ^a^ Pittsburgh sleep quality index, ^b^ Insomnia severity index, ^c^ sleep item of the Roland and Morris disability questionnaire, ^d^ Epworth sleepiness scale.

Table 4 shows the prevalence of insomnia produced by each measure and sensitivity, specificity, positive likelihood ratio (LR^+^) and negative likelihood ratio (LR^-^) with their 95% confidence intervals of the four questionnaires. The global cut-off score of > 5 for the Pittsburgh questionnaire [18] yielded a sensitivity of 100% and a specificity of 44%. The optimal cut-off score of > 6 (generated by the ROC curve technique) resulted in a sensitivity of 100% and a slightly higher specificity of 49% with a LR^+^ of 1.9 and a LR^-^ of 0.0. The prevalence of insomnia was 63%. The cut-off score of > 10 resulted in higher specificity of 85% and a sensitivity of 50%. For the Insomnia index, we used the cut-off point of > 14 as reported in the literature [19] and found it to be the optimal cut-off point. It yielded a sensitivity of 60% and a specificity of 86% and the LR^+^ was 4.43 with 0.46 for the LR. The > 14 cut-off point resulted in an insomnia prevalence of 25%. The Roland item yielded a sensitivity of 49% and a specificity of 80% and the prevalence of insomnia was 58%. Finally, the cut-off score of > 10 for the Epworth scale [26] resulted in sensitivity of 76% with very low specificity (20%) and the prevalence of insomnia was 23%. For details of all scores’ properties of the Pittsburgh questionnaire and the Insomnia index [see Additional file 1 and Additional file 2].

**Table 4:** Prevalence of insomnia, sensitivity, specificity, positive likelihood ratio and negative likelihood ratio values at reported cut-off points and optimal discriminatory cut-off point for each measure

***Test***	***Cut-off point***	***Insomnia %***	***Sensitivity***	***Specificity***	***LR***^***+ a***^	***LR***^***- b***^
			***(95% CI)***	***(95% CI)***	***(95% CI)***	***(95% CI)***
Sleep diary	(ICSD-2)^c^	25%				
Pittsburgh questionnaire^d^	>5	67%	100 (83–100)	44 (31–58)	1.8 (1.3 - 2.4)	0
	**>6**^**e**^	63%	100 (83 – 100)	49 (36 – 63)	1.9 (1.5 – 2.5)	0
	>10	24%	50 (27–73)	85 (73–93)	3.28 (2.1 - 5.1)	0.59 (0.3 - 1.2)
Insomnia index^f^	**>14**^**e**^	25%	60 (36–81)	86 (75–94)	4.43 (3.1 - 6.4)	0.46 (0.2 - 1.1)
Roland item^g^	Yes/no	58%	49 (36 – 63)	80 (56 – 94)	2.46 (0.99 – 6.13)	0.64 (0.46 – 0.89)
Epworth scale^h^	>10	23%	76 (63 – 86)	20 (6 – 44)	0.95 (0.73 – 1.24)	1.19 (0.44 – 3.19)

^a^ Positive likelihood ratio, ^b^ Negative likelihood ratio, ^c^ International classification of sleep disorders, ^d^ Pittsburgh sleep quality index, ^e^ Maximized cut-off point, ^f^ Insomnia severity index, ^g^ Sleep item of the Roland and Morris disability questionnaire, ^h^ Epworth sleepiness scale.

## Discussion

Insomnia is prevalent in patients with LBP [5,9]. Identifying a measure to detect insomnia that is accurate, brief and easy to administer in this population is essential so that clinicians and researchers can gain a more complete understanding of these common co-morbidities. We evaluated the discriminatory properties of four self-report sleep measures (the Pittsburgh questionnaire, Insomnia index, Epworth scale and the Roland item). These measures were selected because they are widely employed to assess sleep quality and they assess some insomnia symptoms such as sleep disturbance and day-time sleepiness. Our findings suggest that the Pittsburgh questionnaire and the Insomnia index are accurate instruments for screening insomnia in patients with LBP, while the Epworth and the Roland have unacceptably low accuracy.

The Epworth scale and Roland item showed poor accuracy to distinguish between patients with insomnia and those without. This may be because each of these questionnaires only assesses a single criterion of insomnia. The Roland item measures sleep quality (the impact of LBP on a patient’s sleep), while the Epworth scale measures day-time sleepiness. Although sleep disturbance and day-time sleepiness are considered to be important traits of insomnia diagnosis, they are inadequate for an insomnia diagnosis.

Although the Pittsburgh questionnaire is designed to assess sleep quality and sleep disturbance, it additionally includes the assessment of day-time impairment related to disturbed sleep: day-time sleepiness and the influence of disturbed sleep on a patient’s social activity. Likewise the Insomnia index measures the severity of insomnia and its effect on a patient’s day-time functioning. The ROC analysis indicated that both the Pittsburgh questionnaire and Insomnia index were able to accurately distinguish patients with insomnia from those without. These findings suggest that when the goal is to diagnose insomnia a measure that combines several insomnia criteria is more accurate than a single criterion measure. This is supported by the work of Sanford et al. [37] who evaluated the accuracy of the Epworth scale to diagnose insomnia in a community sample. They found that although participants with insomnia reported statistically significantly higher scores of sleepiness than those without insomnia, the ROC curve analysis demonstrated that the scale had poor accuracy to diagnose insomnia. The authors concluded that a measure that combines several insomnia criteria is likely to provide a more accurate diagnosis.

Identifying the optimal cut-off score of a questionnaire is essential to identify patients who require further sleep evaluation and those who may require intervention. Our analysis of sensitivity and specificity for the Pittsburgh questionnaire using the recommended global score of > 5 showed that although the questionnaire yielded a high sensitivity of 100%, the specificity was low at 44%. The optimal cut-off score of > 6, identified from the ROC curve, resulted in a slightly higher specificity of 49% with 100% sensitivity. This finding concurs with the finding of Backhaus et al. [18] who examined the psychometric properties of the Pittsburgh questionnaire for patients with primary insomnia. The authors reported that the cut-off score of > 6 increased the questionnaire’s capacity to rule out patients without clinical insomnia. Although both scores yielded a high sensitivity (100%), which indicated that all of the patients with insomnia have been detected, both cut-off scores had low specificity suggesting that half of the patients had been incorrectly identified as having insomnia. Higher sensitivity is an important characteristic of a screening tool in the primary care setting as it is often more important not to miss any patients with the condition than to incorrectly identify some without it. On the other hand, for research purposes high specificity may be required to rule out people without the condition and therefore provide homogenous sample of individuals with a high probably of having the condition. It has been suggested that cut-off score of > 10 for the Pittsburgh questionnaire would increase the questionnaire’s accuracy to detect people who have difficulty in initiating and maintaining sleep, which is an important trait of insomnia [17,36]. Our data showed the cut-off score > 10 resulted in a specificity of 85% with a sensitivity of 50%. The rate of insomnia accordingly declined to 24%, which was closer to the estimate reported by the sleep diary (25%).

The cut-off score of > 14 for the Insomnia index produced optimal discrimination between patients with insomnia and those without (sensitivity 60% and specificity 86%). This result is similar to that of Smith and Trinder [19] who found that a cut-off score of > 14 provided optimal discrimination between people with primary insomnia and the control group. Although this score maximized both sensitivity and specificity to 94%, with an AUC value of 0.97, the study was a case–control design which might have led to overestimation of test accuracy [38]. Savard et al. [14] who examined the discriminatory properties of the Insomnia index in a sample of 1670 patients with cancer, similarly reported that the cut-off score of >14 provided optimal discrimination between patients with insomnia and those without insomnia (sensitivity 51% and specificity 91%).

In clinical practice, likelihood ratios are more useful than sensitivity and specificity for characterising test accuracy [39]. Likelihood ratios indicate likely the test result is in people with the disease compared to those without the disease [40]. The likelihood ratio analysis (Table 4) suggests that the Insomnia index was marginally more accurate than the Pittsburgh questionnaire for detecting patients with insomnia. The analysis demonstrated that a score of 15 points or more on the Insomnia index is 4.43 times more likely in someone with insomnia than someone without insomnia. In addition to its accuracy, the Insomnia index is briefer, easier to administer and to score than the Pittsburgh questionnaire. The Insomnia index therefore appears to be most useful for insomnia assessment of patients with LBP.

This study had limitations that should be addressed. First, although the sleep diary is considered to be a useful tool to diagnose insomnia, its subjective nature is a disadvantage. It has been suggested that patients with sleep problems have the tendency to misperceive their sleep. These patients tend to underestimate their sleep duration and overestimate sleep latency and duration of waking after sleep onset [41]. This limitation potentially influenced sleep variables derived from the sleep diary and therefore the study findings. Second, the study did not investigate sleep-related disorders that may occur along with insomnia, for example sleep apnea and periodic limb movement, which might confound the insomnia assessment. A strength of this study is however that we followed the guidelines for assessing insomnia [1] and the recommendations for designing studies for a diagnostic testing [38]. Additionally, it is the first study to provide guidelines for the optimal assessment of insomnia in patients with LBP using self-reported questionnaires. Finally, the inclusion of patients with LBP who were seeking health care for their LBP, as well as those who were not, increases the representativeness of the sample.

## Conclusions

This is the first study to provide guidelines for the optimal assessment of insomnia in patients with LBP using self-reported questionnaires. The study findings showed that the Pittsburgh questionnaire and the Insomnia index are useful instruments for screening insomnia in patients with LBP. The cut-off score of > 6 for the Pittsburgh questionnaire and cut-off score of > 14 for the Insomnia index provided optimal sensitivity and specificity for the detection of insomnia. The Insomnia index is the briefer, easier to administer and to score, therefore, appears the most appropriate instrument to identify insomnia in the LBP population.

## Competing interests

The authors have no competing interests to report.

## Authors’ contributions

SA was responsible for data collection, data analysis and drafting of the manuscript. JM contributed to the design and conception of the study, critical revision, important intellectual content and drafting of the manuscript. CM helped with the design and conception of the study, the statistics, and also performed critical revisions and supervision. JH contributed to the study design and critical revision. NH was involved in the statistics interpretation of data. DB and RG helped with data collection, data analysis and critical revisions and drafting of the manuscript. All of the authors have read and approved the final manuscript.

## Pre-publication history

The pre-publication history for this paper can be accessed here:

http://www.biomedcentral.com/1471-2474/14/196/prepub

## Supplementary Material

Additional file 1 **Properties of scores of the Pittsburgh questionnaire (doc).** The file provides sensitivity, specificity and positive likelihood ratio and negative likelihood ratio values of the Pittsburgh questionnaire scores. Click here for file

Additional file 2 **Properties of scores of the Insomnia index (doc).** The file provides sensitivity, specificity and positive likelihood ratio and negative likelihood ratio values of the Insomnia index scores. Click here for file

## References

[B1] Edinger JD Bonnet MH Bootzin RR Doghramji K Dorsey CM Espie CA Jamieson AO McCall WV Morin CM Stepanski EJ American Academy of Sleep Medicine Work G Derivation of research diagnostic criteria for insomnia: report of an American Academy of Sleep Medicine Work Group Sleep 2004 27 1567 1596 15683149 10.1093/sleep/27.8.1567

[B2] Summers MO Crisostomo MI Stepanski EJ Recent developments in the classification, evaluation, and treatment of insomnia Chest 2006 130 276 286 10.1378/chest.130.1.276 16840413

[B3] Buysse DJ Ancoli-Israel S Edinger JD Lichstein KL Morin CM Recommendations for a standard research assessment of insomnia Sleep 2006 29 1155 1173 17040003 10.1093/sleep/29.9.1155

[B4] Sivertsen B Krokstad S Overland S Mykletun A The epidemiology of insomnia: associations with physical and mental health. The HUNT-2 study J Psychosom Res 2009 67 109 116 10.1016/j.jpsychores.2009.05.001 19616137

[B5] van de Water AT Eadie J Hurley DA Investigation of sleep disturbance in chronic low back pain: an age- and gender-matched case–control study over a 7-night period Man Ther 2011 16 550 556 10.1016/j.math.2011.05.004 21652257

[B6] O’Donoghue GM Fox N Heneghan C Hurley DA Objective and subjective assessment of sleep in chronic low back pain patients compared with healthy age and gender matched controls: a pilot study BMC Musculoskelet Disord 2009 10 122 10.1186/1471-2474-10-122 19799778 PMC2765952

[B7] Morin CM Benca R Chronic insomnia Lancet 2012 379 1129 1141 10.1016/S0140-6736(11)60750-2 22265700

[B8] Haack M Scott-Sutherland J Santangelo G Simpson NS Sethna N Mullington JM Pain sensitivity and modulation in primary insomnia Eur J Pain 2012 16 522 533 10.1016/j.ejpain.2011.07.007 22396081 PMC3627385

[B9] Tang NKY Wright KJ Salkovskis PM Prevalence and correlates of clinical insomnia co-occurring with chronic back pain J Sleep Res 2007 16 85 95 10.1111/j.1365-2869.2007.00571.x 17309767

[B10] Buysse DJ Reynolds CF 3rd Monk TH Berman SR Kupfer DJ The Pittsburgh Sleep Quality Index: a new instrument for psychiatric practice and research Psychiatry Res 1989 28 193 213 10.1016/0165-1781(89)90047-4 2748771

[B11] Morin C Insomnia: Psychological Assessment and Management 1993 New York, London: The Guilford Press

[B12] Johns MW A new method for measuring daytime sleepiness: the Epworth sleepiness scale Sleep 1991 14 540 545 1798888 10.1093/sleep/14.6.540

[B13] Roland M Morris R A study of the natural history of back pain. Part I: development of a reliable and sensitive measure of disability in low-back pain Spine 1983 8 141 144 10.1097/00007632-198303000-00004 6222486

[B14] Savard MH Savard J Simard S Ivers H Empirical validation of the Insomnia Severity Index in cancer patients Psychooncology 2005 14 429 441 10.1002/pon.860 15376284

[B15] Beck SL Schwartz AL Towsley G Dudley W Barsevick A Psychometric evaluation of the Pittsburgh Sleep Quality Index in cancer patients J Pain Symptom Manage 2004 27 140 148 10.1016/j.jpainsymman.2003.12.002 15157038

[B16] Fictenberg NL Putnam SH Mann NR Zafonte RD Millard AE Insomnia screening in postacute traumatic brain injury: utility and validity of the Pittsburgh Sleep Quality Index Am J Phys Med Rehabil 2001 80 339 345 10.1097/00002060-200105000-00003 11327555

[B17] Violani C Devoto A Lucidi F Lombardo C Russo PM Validity of a short insomnia questionnaire: the SDQ Brain Res Bull 2004 63 415 421 10.1016/j.brainresbull.2003.06.002 15245769

[B18] Backhaus J Junghanns K Broocks A Riemann D Hohagen F Test-retest reliability and validity of the Pittsburgh Sleep Quality Index in primary insomnia J Psychosom Res 2002 53 737 740 10.1016/S0022-3999(02)00330-6 12217446

[B19] Smith S Trinder J Detecting insomnia: comparison of four self-report measures of sleep in a young adult population J Sleep Res 2001 10 229 235 10.1046/j.1365-2869.2001.00262.x 11696076

[B20] Morin CM Belleville G Belanger L Ivers H The Insomnia Severity Index: psychometric indicators to detect insomnia cases and evaluate treatment response Sleep 2011 34 601 608 21532953 10.1093/sleep/34.5.601 PMC3079939

[B21] Monk TH Reynolds CF 3rd Kupfer DJ Buysse DJ Coble PA Hayes AJ Machen MA Petrie SR Ritenour AM The Pittsburgh Sleep Diary J Sleep Res 1994 3 111 120 10.1111/j.1365-2869.1994.tb00114.x 10607115

[B22] Balague F Mannion AF Pellise F Cedraschi C Non-specific low back pain Lancet 2012 379 482 491 10.1016/S0140-6736(11)60610-7 21982256

[B23] Cleeland CS Ryan KM Pain assessment: global use of the Brief Pain Inventory Ann Acad Med Singapore 1994 23 129 138 8080219

[B24] Brown TA Chorpita BF Korotitsch W Barlow DH Psychometric properties of the Depression Anxiety Stress Scales (DASS) in clinical samples Behav Res Ther 1997 35 79 89 10.1016/S0005-7967(96)00068-X 9009048

[B25] Krupp LB LaRocca NG Muir-Nash J Steinberg AD The fatigue severity scale. Application to patients with multiple sclerosis and systemic lupus erythematosus Arch Neurol 1989 46 1121 1123 10.1001/archneur.1989.00520460115022 2803071

[B26] Chervin RD Aldrich MS Pickett R Guilleminault C Comparison of the results of the Epworth Sleepiness Scale and the Multiple Sleep Latency Test J Psychosom Res 1997 42 145 155 10.1016/S0022-3999(96)00239-5 9076642

[B27] Davidson M Rasch analysis of 24-, 18- and 11-item versions of the Roland-Morris Disability Questionnaire Qual Life Res 2009 18 473 481 10.1007/s11136-009-9456-4 19238585

[B28] Kelly GA Blake C Power CK O’Keeffe D Fullen BM The association between chronic low back pain and sleep: a systematic review Clin J Pain 2011 27 169 181 10.1097/AJP.0b013e3181f3bdd5 20842008

[B29] Passik SD Whitcomb LA Kirsh KL Theobald DE An unsuccessful attempt to develop a single-item screen for insomnia in cancer patients J Pain Symptom Manage 2003 25 284 287 10.1016/S0885-3924(02)00692-9 12614963

[B30] Alsaadi SM McAuley JH Hush JM Maher CG Erratum to: Prevalence of sleep disturbance in patients with low back pain Eur Spine J 2012 21 554 560 21863463 10.1007/s00586-011-1954-8 PMC3296846

[B31] Buysse DJ Hall ML Strollo PJ Kamarck TW Owens J Lee L Reis SE Matthews KA Relationships between the Pittsburgh Sleep Quality Index (PSQI), Epworth Sleepiness Scale (ESS), and clinical/polysomnographic measures in a community sample J Clin Sleep Med 2008 4 563 571 19110886 PMC2603534

[B32] Lichstein KL Durrence HH Taylor DJ Bush AJ Riedel BW Quantitative criteria for insomnia Behav Res Ther 2003 41 427 445 10.1016/S0005-7967(02)00023-2 12643966

[B33] Zamora J Abraira V Muriel A Khan K Coomarasamy A Meta-DiSc: a software for meta-analysis of test accuracy data BMC Med Res Methodol 2006 6 31 10.1186/1471-2288-6-31 16836745 PMC1552081

[B34] DeLong ER DeLong DM Clarke-Pearson DL Comparing the areas under two or more correlated receiver operating characteristic curves: a nonparametric approach Biometrics 1988 44 837 845 10.2307/2531595 3203132

[B35] Swets JA Measuring the accuracy of diagnostic systems Science 1988 240 1285 1293 10.1126/science.3287615 3287615

[B36] Okun ML Kravitz HM Sowers MF Moul DE Buysse DJ Hall M Psychometric evaluation of the Insomnia Symptom Questionnaire: a self-report measure to identify chronic insomnia J Clin Sleep Med 2009 5 41 51 19317380 PMC2637165

[B37] Sanford SD Lichstein KL Durrence HH Riedel BW Taylor DJ Bush AJ The influence of age, gender, ethnicity, and insomnia on Epworth sleepiness scores: a normative US population Sleep Med 2006 7 319 326 10.1016/j.sleep.2006.01.010 16713340

[B38] Lijmer JG Mol BW Heisterkamp S Bonsel GJ Prins MH van der Meulen JH Bossuyt PM Empirical evidence of design-related bias in studies of diagnostic tests JAMA 1999 282 1061 1066 10.1001/jama.282.11.1061 10493205

[B39] Sonis J How to use and interpret interval likelihood ratios Fam Med 1999 31 432 437 10367208

[B40] Deeks JJ Altman DG Diagnostic tests 4: likelihood ratios BMJ 2004 329 168 169 10.1136/bmj.329.7458.168 15258077 PMC478236

[B41] Harvey AG Tang NK (Mis)perception of sleep in insomnia: a puzzle and a resolution Psychol Bull 2012 138 77 101 21967449 10.1037/a0025730 PMC3277880

