# Supplementary material for: Detecting insomnia in patients with low back pain: accuracy of four self-report sleep measures
Source: BMC Musculoskelet Disord. 2013 Jun 27;14:196. doi: 10.1186/1471-2474-14-196 (PMC3701511; doi:10.1186/1471-2474-14-196)
Supplement: Additional file 1 — Properties of scores of the Pittsburgh questionnaire (doc). The file provides sensitivity, specificity and positive likelihood ratio and negative likelihood ratio values of the Pittsburgh questionnaire scores. [file 1471-2474-14-196-S1.docx]

| **Additional file 1: prosperities of the Pittsburgh questionnaire scores**. | | | | | | | | |
| --- | --- | --- | --- | --- | --- | --- | --- | --- |
| Score | Sensitivity | 95% CI | Specificity | 95% CI | +LR | 95% CI | -LR | 95% CI |
| >=1 | 100.00 | 83.2 - 100.0 | 0.00 | 0.0 - 6.1 | 1.00 |  |  |  |
| >1 | 100.00 | 83.2 - 100.0 | 1.69 | 0.04 - 9.1 | 1.02 | 0.1 - 7.1 | 0.00 |  |
| >3 | 100.00 | 83.2 - 100.0 | 16.95 | 8.4 - 29.0 | 1.20 | 0.7 - 2.1 | 0.00 |  |
| >4 | 100.00 | 83.2 - 100.0 | 25.42 | 15.0 - 38.4 | 1.34 | 0.9 - 2.1 | 0.00 |  |
| >5 | 100.00 | 83.2 - 100.0 | 44.07 | 31.2 - 57.6 | 1.79 | 1.3 - 2.4 | 0.00 |  |
| **>6 *** | 100.00 | 83.2 - 100.0 | 49.15 | 35.9 - 62.5 | 1.97 | 1.5 - 2.5 | 0.00 |  |
| >7 | 80.00 | 56.3 - 94.3 | 57.63 | 44.1 - 70.4 | 1.89 | 1.4 - 2.6 | 0.35 | 0.1 - 0.9 |
| >8 | 70.00 | 45.7 - 88.1 | 71.19 | 57.9 - 82.2 | 2.43 | 1.7 - 3.4 | 0.42 | 0.2 - 0.9 |
| >9 | 55.00 | 31.5 - 76.9 | 84.75 | 73.0 - 92.8 | 3.61 | 2.4 - 5.4 | 0.53 | 0.2 - 1.1 |
| >10 | 50.00 | 27.2 - 72.8 | 84.75 | 73.0 - 92.8 | 3.28 | 2.1 - 5.1 | 0.59 | 0.3 - 1.2 |
| >11 | 35.00 | 15.4 - 59.2 | 89.83 | 79.2 - 96.2 | 3.44 | 1.9 - 6.3 | 0.72 | 0.3 - 1.6 |
| >12 | 30.00 | 11.9 - 54.3 | 91.53 | 81.3 - 97.2 | 3.54 | 1.8 - 6.9 | 0.76 | 0.3 - 1.9 |
| >13 | 20.00 | 5.7 - 43.7 | 94.92 | 85.9 - 98.9 | 3.93 | 1.6 - 9.5 | 0.84 | 0.3 - 2.6 |
| >14 | 10.00 | 1.2 - 31.7 | 94.92 | 85.9 - 98.9 | 1.97 | 0.5 - 7.3 | 0.95 | 0.3 - 2.9 |
| >15 | 5.00 | 0.1 - 24.9 | 96.61 | 88.3 - 99.6 | 1.47 | 0.2 - 10.0 | 0.98 | 0.3 - 3.9 |
| >16 | 5.00 | 0.1 - 24.9 | 98.31 | 90.9 - 100.0 | 2.95 | 0.4 - 19.9 | 0.97 | 0.1 - 6.8 |
| >18 | 0.00 | 0.0 - 16.8 | 98.31 | 90.9 - 100.0 | 0.00 |  | 1.02 | 0.1 - 7.1 |
| >19 | 0.00 | 0.0 - 16.8 | 100.00 | 93.9 - 100.0 |  |  | 1.00 |  |
| ***** Optimal cut-off score; Pittsburgh questionnaire, Pittsburgh sleep quality index;  +LR, positive likelihood ratio; -LR, negative likelihood ratio. | | | | | | | | |
