# Supplementary material for: Detecting insomnia in patients with low back pain: accuracy of four self-report sleep measures
Source: BMC Musculoskelet Disord. 2013 Jun 27;14:196. doi: 10.1186/1471-2474-14-196 (PMC3701511; doi:10.1186/1471-2474-14-196)
Supplement: Additional file 2 — Properties of scores of the Insomnia index (doc). The file provides sensitivity, specificity and positive likelihood ratio and negative likelihood ratio values of the Insomnia index scores. [file 1471-2474-14-196-S2.docx]

| **Additional file 2: prosperities of the Insomnia index scores.** | | | | | | | | |
| --- | --- | --- | --- | --- | --- | --- | --- | --- |
| Score | Sensitivity | 95% CI | Specificity | 95% CI | +LR | 95% CI | -LR | 95% CI |
| >=0 | 100.00 | 83.2 - 100.0 | 0.00 | 0.0 - 6.1 | 1.00 |  |  |  |
| >0 | 100.00 | 83.2 - 100.0 | 3.39 | 0.4 - 11.7 | 1.04 | 0.3 - 4.0 | 0.00 |  |
| >2 | 100.00 | 83.2 - 100.0 | 5.08 | 1.1 - 14.1 | 1.05 | 0.3 - 3.2 | 0.00 |  |
| >3 | 100.00 | 83.2 - 100.0 | 10.17 | 3.8 - 20.8 | 1.11 | 0.5 - 2.4 | 0.00 |  |
| >4 | 100.00 | 83.2 - 100.0 | 15.25 | 7.2 - 27.0 | 1.18 | 0.6 - 2.2 | 0.00 |  |
| >5 | 100.00 | 83.2 - 100.0 | 27.12 | 16.4 - 40.3 | 1.37 | 0.9 - 2.1 | 0.00 |  |
| >6 | 100.00 | 83.2 - 100.0 | 33.90 | 22.1 - 47.4 | 1.51 | 1.1 - 2.2 | 0.00 |  |
| >7 | 95.00 | 75.1 - 99.9 | 37.29 | 25.0 - 50.9 | 1.51 | 1.1 - 2.1 | 0.13 | 0.02 - 0.9 |
| >8 | 90.00 | 68.3 - 98.8 | 49.15 | 35.9 - 62.5 | 1.77 | 1.3 - 2.4 | 0.20 | 0.05 - 0.8 |
| >9 | 80.00 | 56.3 - 94.3 | 57.63 | 44.1 - 70.4 | 1.89 | 1.4 - 2.6 | 0.35 | 0.1 - 0.9 |
| >10 | 75.00 | 50.9 - 91.3 | 61.02 | 47.4 - 73.5 | 1.92 | 1.4 - 2.7 | 0.41 | 0.2 - 0.9 |
| >11 | 70.00 | 45.7 - 88.1 | 66.10 | 52.6 - 77.9 | 2.06 | 1.5 - 2.9 | 0.45 | 0.2 - 1.0 |
| >12 | 65.00 | 40.8 - 84.6 | 72.88 | 59.7 - 83.6 | 2.40 | 1.7 - 3.4 | 0.48 | 0.2 - 1.0 |
| >13 | 60.00 | 36.1 - 80.9 | 83.05 | 71.0 - 91.6 | 3.54 | 2.4 - 5.2 | 0.48 | 0.2 - 1.0 |
| **>14 *** | 60.00 | 36.1 - 80.9 | 86.44 | 75.0 - 94.0 | 4.43 | 3.1 - 6.4 | 0.46 | 0.2 - 1.1 |
| >15 | 45.00 | 23.1 - 68.5 | 88.14 | 77.1 - 95.1 | 3.79 | 2.3 - 6.2 | 0.62 | 0.3 - 1.4 |
| >17 | 40.00 | 19.1 - 63.9 | 89.83 | 79.2 - 96.2 | 3.93 | 2.3 - 6.8 | 0.67 | 0.3 - 1.5 |
| >18 | 40.00 | 19.1 - 63.9 | 91.53 | 81.3 - 97.2 | 4.72 | 2.7 - 8.1 | 0.66 | 0.3 - 1.6 |
| >19 | 40.00 | 19.1 - 63.9 | 93.22 | 83.5 - 98.1 | 5.90 | 3.4 - 10.1 | 0.64 | 0.2 - 1.8 |
| >20 | 30.00 | 11.9 - 54.3 | 93.22 | 83.5 - 98.1 | 4.42 | 2.3 - 8.7 | 0.75 | 0.3 - 2.0 |
| >21 | 20.00 | 5.7 - 43.7 | 93.22 | 83.5 - 98.1 | 2.95 | 1.2 - 7.1 | 0.86 | 0.3 - 2.3 |
| >22 | 15.00 | 3.2 - 37.9 | 94.92 | 85.9 - 98.9 | 2.95 | 1.0 - 8.4 | 0.90 | 0.3 - 2.7 |
| >23 | 10.00 | 1.2 - 31.7 | 94.92 | 85.9 - 98.9 | 1.97 | 0.5 - 7.3 | 0.95 | 0.3 - 2.9 |
| >24 | 10.00 | 1.2 - 31.7 | 98.31 | 90.9 - 100.0 | 5.90 | 1.6 - 22.0 | 0.92 | 0.1 - 6.4 |
| >25 | 5.00 | 0.1 - 24.9 | 100.00 | 93.9 - 100.0 |  |  | 0.95 |  |
| >28 | 0.00 | 0.0 - 16.8 | 100.00 | 93.9 - 100.0 |  |  | 1.00 |  |
| ***** optimal cut-off score; Insomnia index, insomnia severity index;  +LR, positive likelihood ratio; -LR, negative likelihood ratio | | | | | | | | |
